# The impact of postoperative calcaneocuboid joint articular surface gap and step on long-term outcomes in surgically treated calcaneal fractures: a brief report

**DOI:** 10.1186/s12891-026-10085-8

**Published:** 2026-06-11

**Authors:** Sayyed-Hadi Sayyed-Hosseinian, Sadaf Samadi, Parisa Yazdi, Majid Ghodsi, Matin Shirazinia, Farshid Bagheri, Mohammad H. Ebrahimzadeh, Amir Abderam

**Affiliations:** 1 Orthopedic Research Center, Shahid Kamyab Hospital, Mashhad University of Medical Sciences, Mashhad, Iran; 2 Faculty of Medicine, Mashhad University of Medical Sciences, Mashhad, Iran; 3 Student Research Center, School of Medicine, Birjand University of Medical Sciences, Ghaffari street, Birjand, South Khorasan Province Iran; 4 Faculty of Medicine, Mashhad Medical Sciences Branch, Islamic Azad University, Mashhad, Iran; 5 Orthopedic Research Center, Ghaem Hospital, Mashhad University of Medical Sciences, Mashhad, Iran

**Keywords:** Calcaneal fractures, Calcaneocuboid joint, Anatomic reduction, Functional outcomes, Reoperation risk

## Abstract

**Background:**

Calcaneal fractures, the most common tarsal fractures, often result in significant long-term disability and impose substantial healthcare burdens. Surgical intervention aims to restore hindfoot anatomy, but the role of calcaneocuboid joint reduction quality in determining long-term outcomes remains unclear. This study evaluated the impact of calcaneocuboid joint reduction quality on functional outcomes and the need for reoperation in surgically treated patients with calcaneal fractures.

**Methods:**

This retrospective cohort study included 42 adult patients who underwent surgical treatment for calcaneal fractures. Postoperative computed tomography (CT) scans were analyzed to assess calcaneocuboid joint parameters, specifically gap and step. Functional outcomes were measured using the Manchester Oxford Foot Questionnaire (MOXFQ), and patient interviews were conducted to document the need for reoperations during the follow-up period.

**Results:**

The median follow-up duration was 6.5 years. Neither the postoperative gap nor step of the calcaneocuboid joint significantly correlated with MOXFQ total scores (gap: Spearman’s *r* = 0.15, *P* = 0.336; step: Spearman’s *r* = 0.19, *P* = 0.239). Additionally, no significant differences in MOXFQ scores were observed between patients with anatomic and non-anatomic reductions (*P* = 0.548). However, non-anatomic calcaneocuboid joint reduction was significantly associated with a 4.44-fold increased hazard of reoperation in this cohort (hazard ratio: 4.44, 95% CI: 1.48 to 13.31, *P* = 0.008).

**Conclusion:**

While calcaneocuboid joint reduction quality did not significantly affect functional outcomes, the observed association with an increased hazard of reoperation highlights the need for further research to validate these findings and explore potential clinical implications.

## Introduction

Calcaneal fractures, which are the most common tarsal fractures, account for about 1–2% of all bone fractures. The occurrence rate for calcaneal fractures is approximately 11.5–13.7 cases per 100,000 people per year. These fractures most commonly result from falls, motor vehicle accidents, or sports injuries [1–5]. The fracture is often accompanied by permanent or temporary disabilities, creating a burden for both patients and the healthcare system. This is primarily due to changes in the bone structure of the joint surfaces, resulting in reduced height, altered width, and misalignment. Over time, these changes lead to calcaneal malunion and osteoarthritis, which impair activities such as walking, jumping, and standing [6].

Generally, therapeutic options include non-surgical and surgical treatments. Choosing the best approach remains controversial and depends on factors such as the patient’s functional needs, fracture type, and any concurrent injuries. Extensile lateral approach (ELA) and sinus tarsi approach (STA) are among the most frequently used surgical approaches [6]. When surgery is performed, achieving anatomic reduction is crucial. Restoring the hindfoot anatomy after intra-articular calcaneal fractures has increasingly been recognized as a critical factor in obtaining favorable clinical outcomes [7–9]. We previously demonstrated the impact of anatomic reduction of the posterior facet on the functional outcomes of patients, whether treated using ELA or STA. Anatomic reduction was associated with better functional outcomes [10]. While prior studies have investigated the impact of the calcaneocuboid joint on patient outcomes [9, 11], there is limited information in the literature regarding the significance of calcaneocuboid joint reduction quality in long-term patient outcomes [9].

In this study, we aimed to examine the effect of postoperative calcaneocuboid joint reduction quality on the long-term functional outcomes and the need for reoperation in patients with calcaneal fractures treated surgically using STA or ELA.

## Methods

### Study population and study design

A retrospective list of patients who underwent surgery between March 2015 and March 2019 at our centers was compiled. Adult patients with calcaneal fractures (Sander’s types II, III, and IV) who were treated surgically using either the ELA or STA approach were included. Patients were excluded if they were under 18 years of age, the calcaneocuboid joint was not involved, they had Sander’s type I fractures, underwent surgical treatment with other approaches, had a follow-up period of less than five years from the primary operation, post-operative computed tomography (CT) scan was not performed, or were treated conservatively. The details of the surgical methods were described previously in our prior work [10].

### Parameters of calcaneocuboid joint on postoperative CT scans

We collected pre- and post-operative CT scans of patients from the hospital’s picture archiving and communication system (PACS). The gap and step of the calcaneocuboid joint were measured. An anatomic reduction was defined as a remaining gap and step of the calcaneocuboid joint measuring less than 2 mm. If this criterion was not met, the reduction quality was classified as non-anatomic [9]. To maximize measurement reliability, an experienced foot and ankle surgeon assessed all images.

### Measurements at follow-up

Patients were contacted to inquire about their outcomes. For functional outcomes, the Manchester Oxford Foot Questionnaire (MOXFQ) was sent to them via an electronic link [12]. Patients could ask their questions about the MOXFQ items from a member of the project team. The MOXFQ comprises three domains: walking/standing, social interaction, and pain. The total score is calculated out of 100, with a higher score indicating worse functionality. During the calls, patients were verbally asked if they had required any reoperation in the past years and, if so, the timing of the reoperation relative to their first operation.

### Statistical analysis

Statistical analysis was performed using R version 4.3.1 and STATA 14.2. Categorical data were described as numbers and percentages, while continuous data were presented as medians (25th to 75th percentile) for non-normally distributed variables or means (standard deviations) for normally distributed variables. Normality was assessed using the Shapiro-Wilk test. The Mann-Whitney U test or independent t-test was applied to compare MOXFQ scores between groups for non-normal and normal data, respectively. The association between the quantity of the remaining postoperative gap or step in the calcaneocuboid joint with functional outcomes was assessed using correlation coefficients. Survival analysis was conducted using the nonparametric log-rank test and the semiparametric Cox proportional hazards regression to evaluate the effect of various calcaneocuboid joint features on the need for reoperation. The hazard ratio (HR) and 95% confidence interval (95% CI) were reported. All patients were censored at their last follow-up. Additionally, the Kaplan-Meier estimator was used to evaluate survival over the study period. The proportional hazards assumption of the Cox regression was tested, with significant results indicating a violation of the assumption. A two-tailed P-value < 0.05 was considered statistically significant throughout the study.

### Ethical considerations

The Ethics Committee of Mashhad University of Medical Sciences approved the study protocol (IR.MUMS.MEDICAL.REC. 1400.248). The present study was designed and conducted in accordance with the Declaration of Helsinki. Informed consent was obtained from all patients.

## Results

### Patients’ characteristics

A total of 42 patients responded to our invitation and participated in the study. The median age of the patients was 37.5 years (25th to 75th percentile, 30 to 45), with 36 (86%) being male (Table 1). The surgical approach was ELA for 35 patients (83%), while 7 (17%) were treated using STA.

**Table 1:** Characteristics of patients with calcaneus fractures

	Total (*N* = 42)
Age (years), mean (standard deviation)	38.5 (9.9)
Gender, male %	36 (86%)
Comorbidities, %	
Cardiovascular diseases	2 (5)
Cerebrovascular Accident	1 (2)
Mechanism of trauma, %	
Fall from height	30 (71)
Motor vehicle accident	12 (29)
Affected limb, %	
Right	27 (64)
Left	15 (36)
Time from injury to surgery (days), median (percentile 25 to percentile 75)	5.0 (2.0 to 8.0)
Type of fracture, %	
Type 2 A	11 (26)
Type 2B	4 (10)
Type 2 C	2 (4)
Type 3AB	15 (36)
Type 3AC	6 (14)
Type 3BC	0
Type 4	4 (10)

### Follow-up visits

Of all the patients, six (14%) had malunion, and nonunion was observed in one patient. The median follow-up time was 6.5 years (25th to 75th percentile: 6 to 7.4 years). Thirteen patients (31%) underwent reoperation, with one requiring more than one surgery. The types of reoperations included hardware removal in 11 patients (85%) and subtalar arthrodesis in two patients (15%). The median MOXFQ score was 47.7 (25th to 75th percentile: 14.1 to 67.2). The overall median calcaneocuboid joint gap and step were 0.0 millimeters (25th to 75th percentile: 0 to 1.42 mm, range: 0 to 5.48 mm) and 0.0 millimeters (25th to 75th percentile: 0.0 to 0.0, range: 0 to 6.21 mm), respectively.

### Effect of calcaneocuboid joint parameters on MOXFQ score

**Fig. 1:**
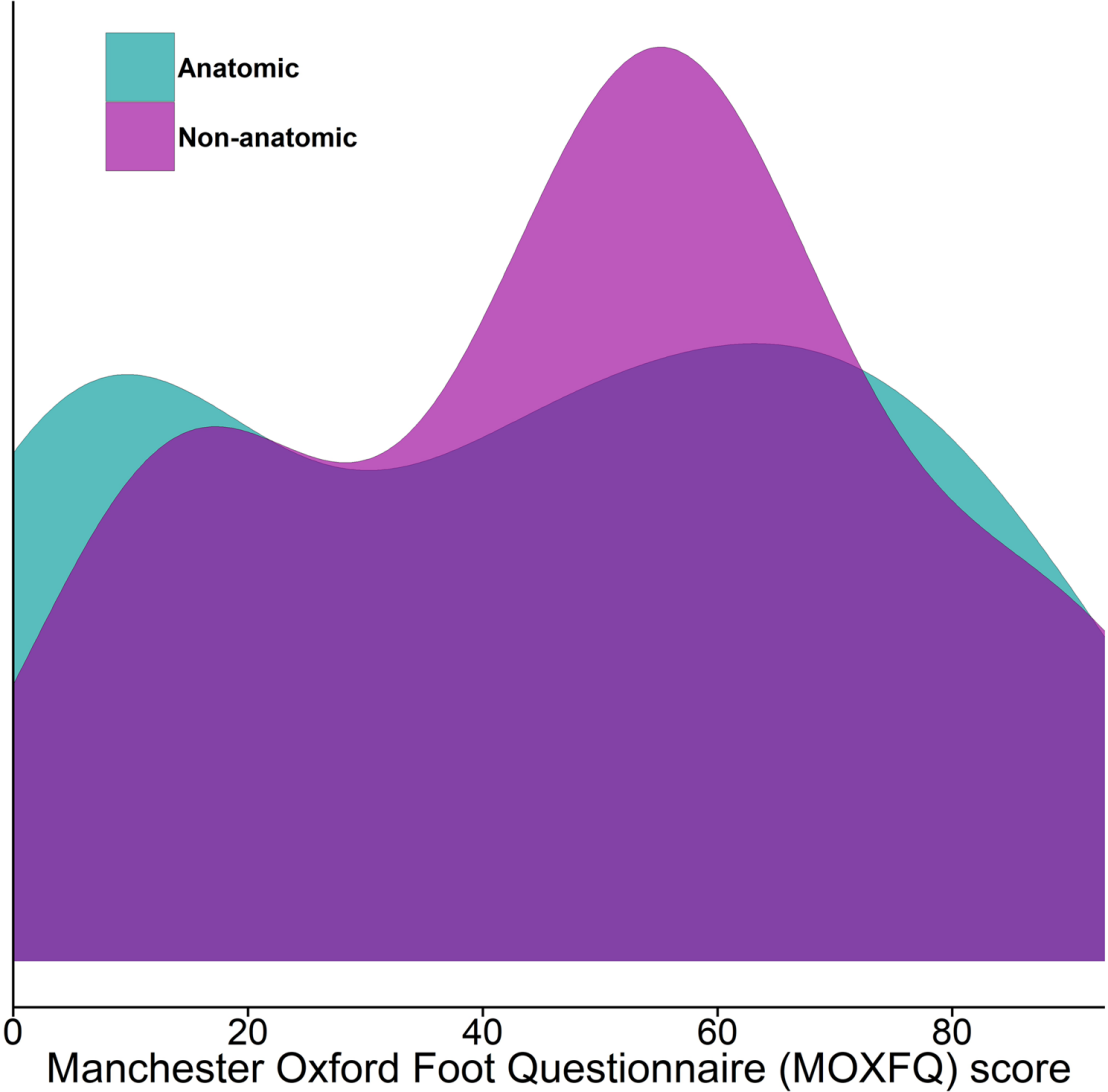
Density plots of manchester oxford foot questionnaire scores comparing anatomic and non-anatomic reductions of the calcaneocuboid joint

Gap (Spearman’s correlation coefficient: 0.15, *P* = 0.336) and step (Spearman’s correlation coefficient: 0.19, *P* = 0.239) showed no significant correlation with the MOXFQ total score. Furthermore, a comparison between anatomic (median: 43.8, 25th to 75th percentile: 12.5 to 70.3) and non-anatomic (median: 51.6, interquartile range: 18.8 to 62.5) calcaneocuboid joint reductions revealed no significant differences in MOXFQ scores (Standardized mean difference: -0.22, 95% CI: -0.89 to 0.46, *P* = 0.548) (Fig. 1).

### Effect of calcaneocuboid joint parameters on the need for reoperation

**Fig. 2:**
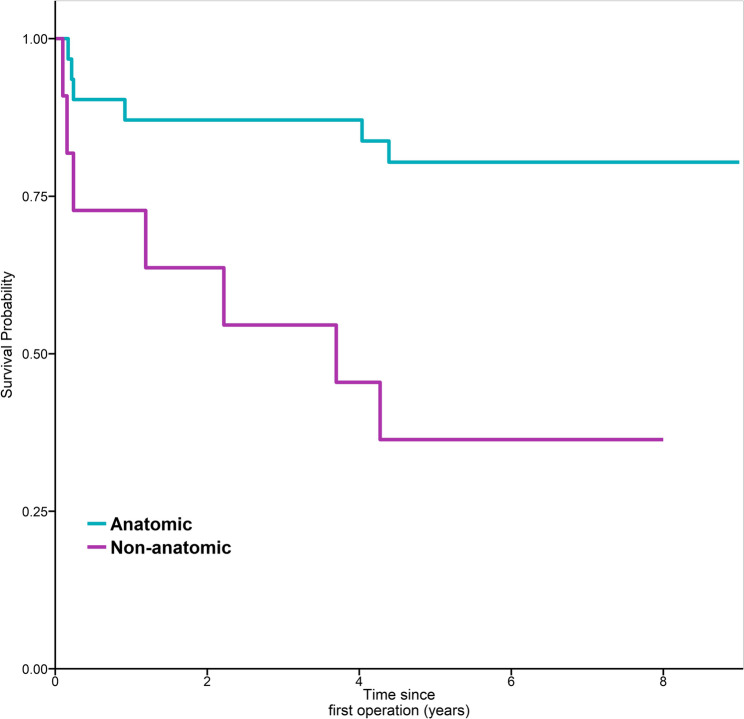
Kaplan-Meier survival curves comparing the need for reoperation between two groups: patients with anatomic reductions and those with non-anatomic reductions of the calcaneocuboid joint

Gap (Hazard ratio [HR]: 1.38, 95% CI: 1.06 to 1.81, *P* = 0.018) was significantly associated with the need for reoperation, whereas step was not significantly associated with reoperation (HR: 0.99, 95% CI: 0.63 to 1.55, *P* = 0.957). Additionally, a non-anatomic calcaneocuboid joint reduction was linked to a 4.44-fold increase in the hazard of reoperation during the follow-up period (HR: 4.44, 95% CI: 1.48 to 13.31, log-rank test *P* = 0.004) (Fig. 2).

## Discussion

The present study assessed the long-term outcomes of 42 patients who underwent surgery for calcaneal fracture treatment. Our findings revealed that the postoperative gap and step of the calcaneocuboid joint did not significantly correlate with patients’ functional outcomes. Furthermore, there was no significant difference in functional outcomes between patients with anatomic and non-anatomic reductions of the calcaneocuboid joint.

In the past, calcaneal fractures were primarily addressed through non-surgical methods. The conservative approach was traditionally believed to be associated with complications such as heel deformities and arthritis. With the progress of surgical techniques, operative treatment has gained popularity [13–15]. However, a large randomized controlled trial (the UK Heel Fracture Trial) including 151 patients comparing operative (ELA) and conservative treatment for intra-articular calcaneal fractures challenged these previous beliefs. Based on the findings of this trial, While there were no significant differences between the two arms in any functional outcomes at a 2-year follow-up, the rate of complications was significantly higher in the surgical group (23% in the ELA group vs. 4% in the conservative group) [16]. An extension of this trial with a 5-year follow-up also showed a significantly increased need for additional operations in the operative group [17]. However, the trial had a pragmatic design, and drawing conclusions regarding non-inferiority or equivalence requires specifically designed studies. Moreover, ELA is not the only surgical option, and there are concerns regarding potential selection bias in this trial [18]. Therefore, the dilemma will continue, and a one-size-fits-all approach is not possible. Decisions should be made based on fracture anatomy, the patient’s underlying conditions, the presence and extent of soft tissue injury, and functional demands [19, 20].

The structure of the hindfoot is intricate, making the treatment of fractures more challenging [21]. When surgery is performed, achieving anatomic reduction is considered essential to prevent long-term complications. Various aspects must be addressed in terms of the anatomic approach, including calcaneal bone height, Böhler’s angle, and the step and gap of the posterior facet and calcaneocuboid joint, to name a few [22]. Previous studies have shown that achieving an anatomic reduction of the posterior facet is associated with better functional outcomes in patients with calcaneal fractures treated surgically [10, 22]. However, not all studies have demonstrated a beneficial impact of anatomic reduction of the posterior facet on functional outcomes [23]. The information regarding this association with the reduction of calcaneocuboid joint is scarce in the literature.

Our study revealed that neither the step nor the gap in the calcaneocuboid joint was significantly correlated with MOXFQ scores during long-term follow-up. Initially, this could be attributed to the small sample size of patients. However, it is important to note that the point estimation of these associations fell within the range of a weak effect [24]. Furthermore, a comparison of MOXFQ scores between anatomic and non-anatomic reductions of the calcaneocuboid joint showed no significant difference between the two groups, with the point estimation of the standardized mean difference (SMD) also falling within the range of a trivial effect [25]. The study conducted by Kinner et al. demonstrated that calcaneocuboid joint involvement in calcaneal fractures did not significantly reduce the American Orthopaedic Foot & Ankle Society (AOFAS) score compared to fractures without calcaneocuboid joint involvement [9]. However, they found significant correlations between the postoperative gap or step of the calcaneocuboid joint and two outcomes: the AOFAS Hindfoot score and the SF-12 score. While these associations were statistically significant, it is important to note that the effect sizes were weak, with a correlation of 0.34 for the AOFAS Hindfoot score and 0.35 for the SF-12 score [24]. Second, they employed a relatively high-powered statistical test (correlation coefficient), meaning the significant result might reflect the nature of the test rather than the strength of the association. Third, the presence of multiple testing in their study increases the likelihood of random error, which could account for the significant results.

The findings of the present study indicated that non-anatomic reduction of the calcaneocuboid joint was associated with a four-fold increased hazard of reoperation during the study period, with the majority of reoperations involving hardware removal due to pain. One possible explanation is that non-anatomic reduction of the calcaneocuboid joint may lead to subtle alterations in hindfoot biomechanics and load distribution, potentially increasing minor stress or motion around the implanted hardware. Although this may suggest a potential relationship between post-operative anatomic reduction and hardware-related pain, caution is warranted. We did not evaluate pain outcomes before and after hardware removal, and therefore cannot conclude that removal resulted in pain relief. We were also unable to assess whether the calcaneocuboid joint was the primary source of pain in symptomatic patients. Image-guided diagnostic injections of local anesthetic into the calcaneocuboid joint could help isolate the joint as a pain source and clarify its clinical importance; however, this was not performed in the present study. Moreover, hardware removal does not address the underlying malreduction. In addition, only calcaneocuboid joint alignment was assessed in this study, while other potential factors (such as surgical approaches) contributing to reoperation were not considered.

This study is among the first to address the importance of postoperative calcaneocuboid joint status in the long-term outcomes of patients with calcaneal fractures. However, it has several limitations. First, the high number of patients without postoperative CT scans, as it is not routine practice, likely introduced selection bias into the results. Second, follow-up was conducted exclusively via phone contact, which excluded radiologic outcomes from the findings. Third, due to the absence of in-person visits, physical examinations could not be performed, resulting in a lack of this crucial information. Fourth, the final sample size was relatively small, limiting the statistical power of the conclusions, which is why we did not employ multivariable analysis to control potential covariates and confounders. Fifth, the current study was a retrospective cohort study and had the inherent limitations of retrospective studies, including limited ability to establish causal relationships. Taken together, these factors suggest that our findings should be interpreted with caution and considered a preliminary evaluation, serving as a foundation for future well-designed prospective studies.

## Conclusion

This study demonstrates that the quality of calcaneocuboid joint reduction does not significantly influence long-term functional outcomes in patients surgically treated for calcaneal fractures. However, non-anatomic reduction of the calcaneocuboid joint was associated with an increased hazard of reoperation. These findings might highlight the complexity of addressing hindfoot fractures and suggest that while functional outcomes might not be heavily affected by calcaneocuboid joint reduction quality, careful attention to anatomic reduction remains possibly crucial to minimize reoperation rates. Further large-scale, prospective studies are needed to validate these results and provide a clearer understanding of their clinical implications.

## Data Availability

The data underlying this article will be shared upon reasonable request to the corresponding author.

## Abbreviations

ELA: Extensile Lateral Approach
STA: Sinus Tarsi Approach
CT: Computed Tomography
PACS: Picture Archiving and Communication System
MOXFQ: Manchester Oxford Foot Questionnaire
HR: Hazard ratio
CI: Confidence Interval
mm: millimeter
SD: Standard Deviation
IQR: Interquartile Range
